# A *Plasmodium falciparum* Strain Expressing GFP throughout the Parasite's Life-Cycle

**DOI:** 10.1371/journal.pone.0009156

**Published:** 2010-02-10

**Authors:** Arthur M. Talman, Andrew M. Blagborough, Robert E. Sinden

**Affiliations:** Division of Cell and Molecular Biology, Imperial College London, London, United Kingdom; Federal University of São Paulo, Brazil

## Abstract

The human malaria parasite *Plasmodium falciparum* is responsible for the majority of malaria-related deaths. Tools allowing the study of the basic biology of *P. falciparum* throughout the life cycle are critical to the development of new strategies to target the parasite within both human and mosquito hosts. We here present 3D7HT-GFP, a strain of *P. falciparum* constitutively expressing the Green Fluorescent Protein (GFP) throughout the life cycle, which has retained its capacity to complete sporogonic development. The GFP expressing cassette was inserted in the *Pf47* locus. Using this transgenic strain, parasite tracking and population dynamics studies in mosquito stages and exo-erythrocytic schizogony is greatly facilitated. The development of 3D7HT-GFP will permit a deeper understanding of the biology of parasite-host vector interactions, and facilitate the development of high-throughput malaria transmission assays and thus aid development of new intervention strategies against both parasite and mosquito.

## Introduction

The human malaria parasite *Plasmodium falciparum* is responsible for the vast majority of malaria-related mortality. Studies of vertebrate-to-vector and vector-to-vertebrate transmissions are of great importance, not only to understand parasite biology but also to aid development of future intervention strategies.

Fluorescent proteins have been extensively used to study malaria parasites and their interactions with host and vector [1]. In particular, examination of sporogonic development and exo-erythrocytic schizogony has made use of *Plasmodium berghei* and *P. yoelii* rodent parasites constitutively expressing the green fluorescent protein (GFP) [2], [3], these studies have considerably contributed to our knowledge of the complex life cycle of malaria parasites [4]. However the significant differences observed between rodent parasites and *P. falciparum*, both at the phylogenetic and biological levels [5], [6], limit the use of these models in many applications. For instance, gametocytogenesis and exo-erythrocytic development are significantly longer for *P. falciparum* [7]. In-vector dynamics also differ greatly, with *P. falciparum* commonly producing fewer oocysts in the mosquito, both in the field and under laboratory conditions [8], [9].

Altogether, these facts stress the need to investigate systematically whether the data generated in mouse models is relevant in a clinical setting. One step in this direction is the establishment of new tools and techniques that allow the easy and efficient tracking and identification of the *P. falciparum* parasite throughout its life stages. We here present the development of a stable transgenic *P. falciparum* parasite that constitutively expresses recombinant green fluorescent protein (GFP) in all life stages, thus facilitating observations in parasite biology and the understanding of intervention measures.

## Results and Discussion

A difficulty with *P. falciparum* transgenesis is the lengthy selection procedure it involves and the tendency of the parasite to lose its ability to infect the mosquito vector within this period [10]. For this reason we have favored integrating the GFP-expressing cassette in the genome by the more rapid method of single homologous recombination.

**Figure 1:**
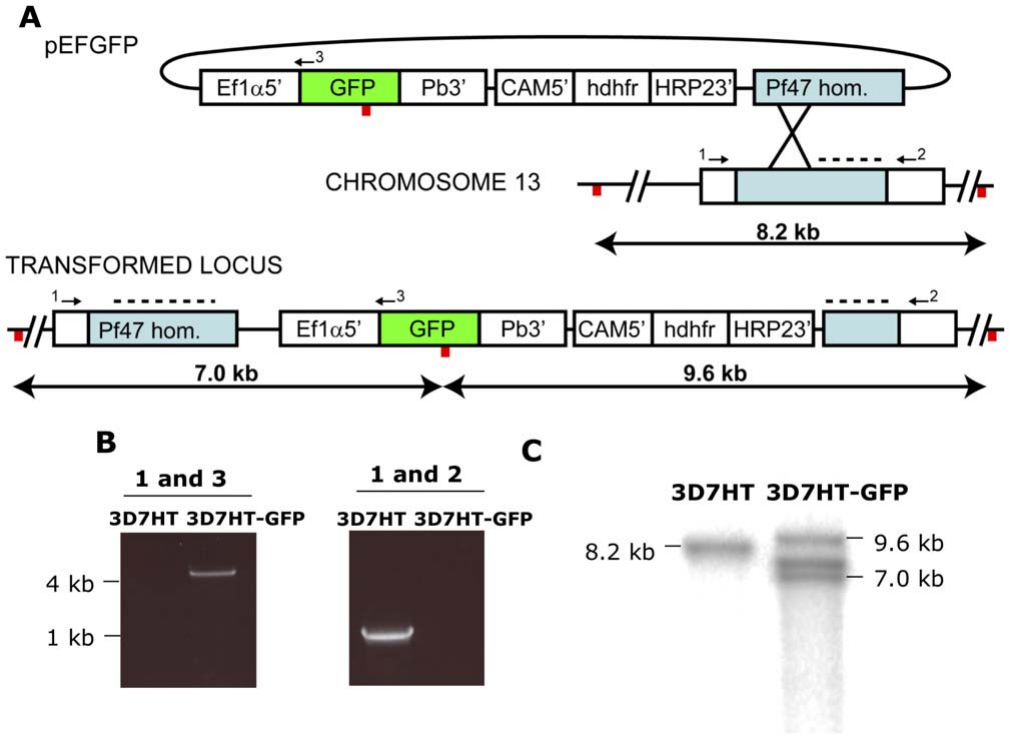
Generation of 3D7HT-GFP. (A) Schematic representation of the plasmid used in this study and integration strategy into *P. falciparum* chromosome 13. pEFGFP contains the Green Fluorescent Protein (GFP) under the control of 800 bp of the EF1 α promoter and the *P.berghei* dhfr 3′ UTR. A 900 bp homology region of the *Pf47* gene is also present. Primers 1 (5′-CAACCCTACGTTGGGTGACC-3′), 2 (5′-TGCGATATGTAATTCCATTACTGC-3′) and 3 (5′-AACAGGTAGTTTTCCAGTAGTGC-3′) were used to monitor the integration event and purity of the transgenic population by PCR, as shown in (B). (C) genomic DNA was digested with HpaI (red bars in (A)), and Southern blot was carried out as described before [17]. The probe was amplified with primers 5′-GGGGCGCCGAATCTCATTTATTCTGC-3′ and 5′-GGGGCGCCTAACATATACAGCCTTCC-3′ and is represented by a black dashed line in (A), The 8.2 kb band for wild type (WT) and 7.0 kb and 9.6 kb bands for 3D7-GFP are detected as expected, and the additional 7.5 kb band indicates the presence of concatamerised integrated copies of the plasmid.

An 800 bp fragment of the *P. falciparum* EF1α (PF13_0304) promoter region was inserted into the pARL plasmid (courtesy of Alan F. Cowman, WEHI). GFP (already present in the parental vector) was therefore placed under the control of the EF1 α promoter and the *P. berghei dhfr* 3′ UTR (Fig. 1). A 900 bp homology region of the *Pf47* coding sequence (PF13_0248) was next inserted into the plasmid, thus obtaining pEFGFP (Fig. 1). The *Pf47* locus was chosen to be the homologous recombination target as it was previously shown to be dispensable for parasite infectivity to mosquitoes [11].

pEFGFP was transfected into an isolate of *P. falciparum* 3D7 recently passaged through the entire life cycle [12], (here termed 3D7-HT). Transfectants were selected and cloned by limiting dilution. The clonal 3D7HT-GFP population was tested for integration of pEFGFP in the *Pf47* locus of chromosome 13 by PCR, and was further validated by Southern blotting (Fig. 1). The length of the entire procedure, from transfection to obtaining the 3D7HT-GFP clone was just 90 days.

**Figure 2:**
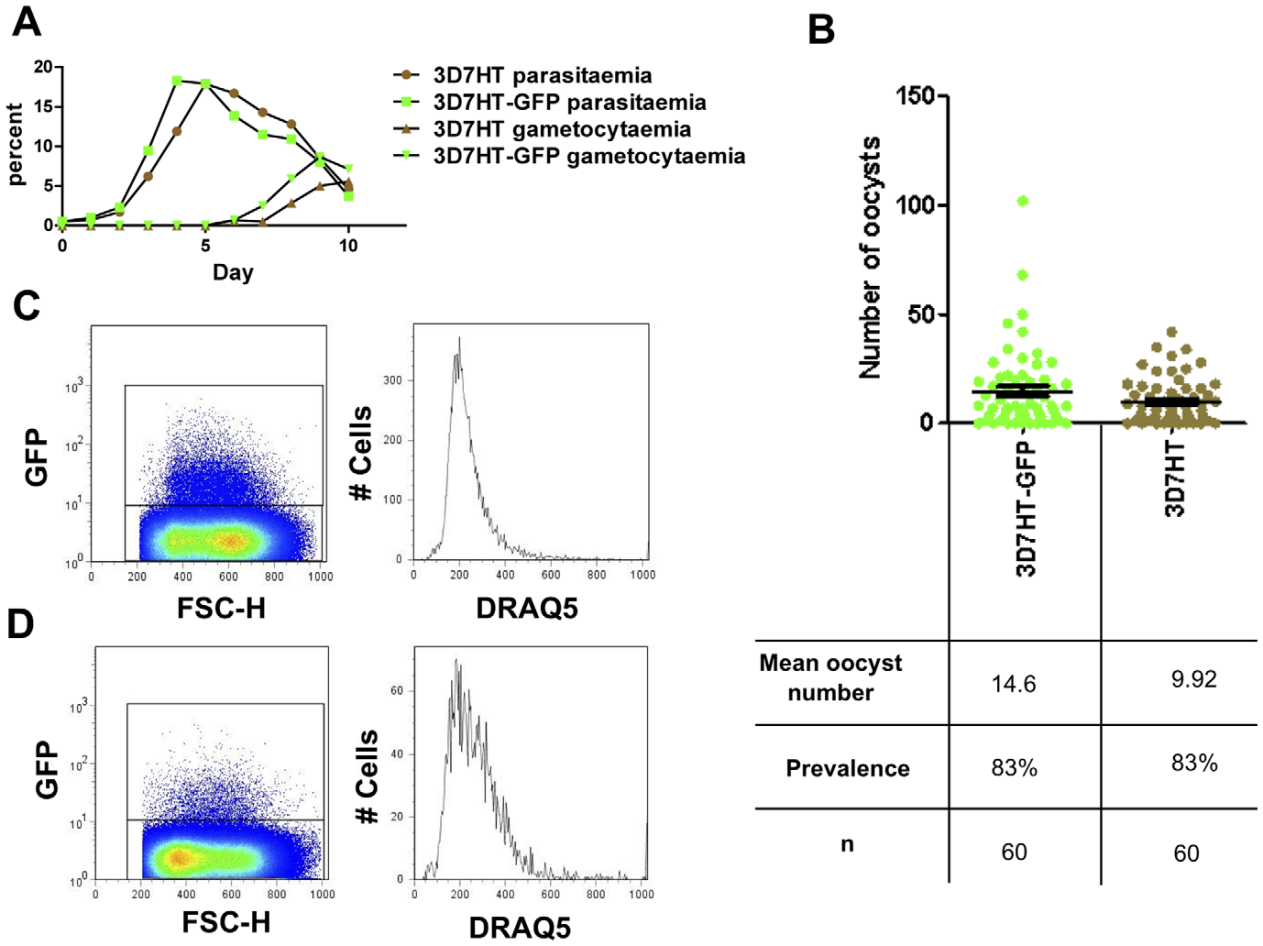
3D7HT-GFP has retained the parental (3D7HT) features. (A) Asexual growth and gametocyte production were measured in 3D7HT and 3D7HT-GFP, means of three independent experiments are plotted. (B) Parasite infectivity to *An. stephensi* was assessed in two independent feeds; oocyst numbers per dissected midgut were recorded. Asexual parasite culture (containing mostly ring stages) (C) and gametocyte culture (D) were analyzed by flow cytometry. GFP-positive events are shown on the left panel, the GFP positive events were further assessed for nuclear content (using DRAQ 5 nuclear stain). The typical higher DNA content of gametocytes can be readily observed [18].

We further verified that transgenesis did not alter the fitness of the parasite as compared to that of the parent strain. Asexual growth rate and gametocyte production in the two strains were indistinguishable (Fig. 2) (P = 0.9219 and P = 0.1250 respectively by Wilcoxon signed rank test). The infectivity of 3D7HT-GFP to mosquitoes was assessed. In two independent mosquito feeds midgut infections were scored for oocyst numbers at day 10. Both 3D7HT and 3D7HT-GFP strains were found to be equally infectious to *Anopheles stephensi* (P = 0.2544, Mann Whitney test) (Fig. 2). This result is consistent with the studies of van Schaijk and colleagues [11], which found the *Pf47* locus dispensable to parasite infectivity to the vector. 3D7HT-GFP oocyst fluorescence was also observed in *An. gambiae* (N'gousso strain, data not shown).

Whilst recognizing the potential for the loss of the GFP encoding insert, no genetic or phenotypic evidence of reversion of 3D7HT-GFP has been found after 4 months continuous culture without drug pressure. In none of the full repertoire of 3D7HT-GFP life stages, did we observe a non-fluorescent parasite.

**Figure 3:**
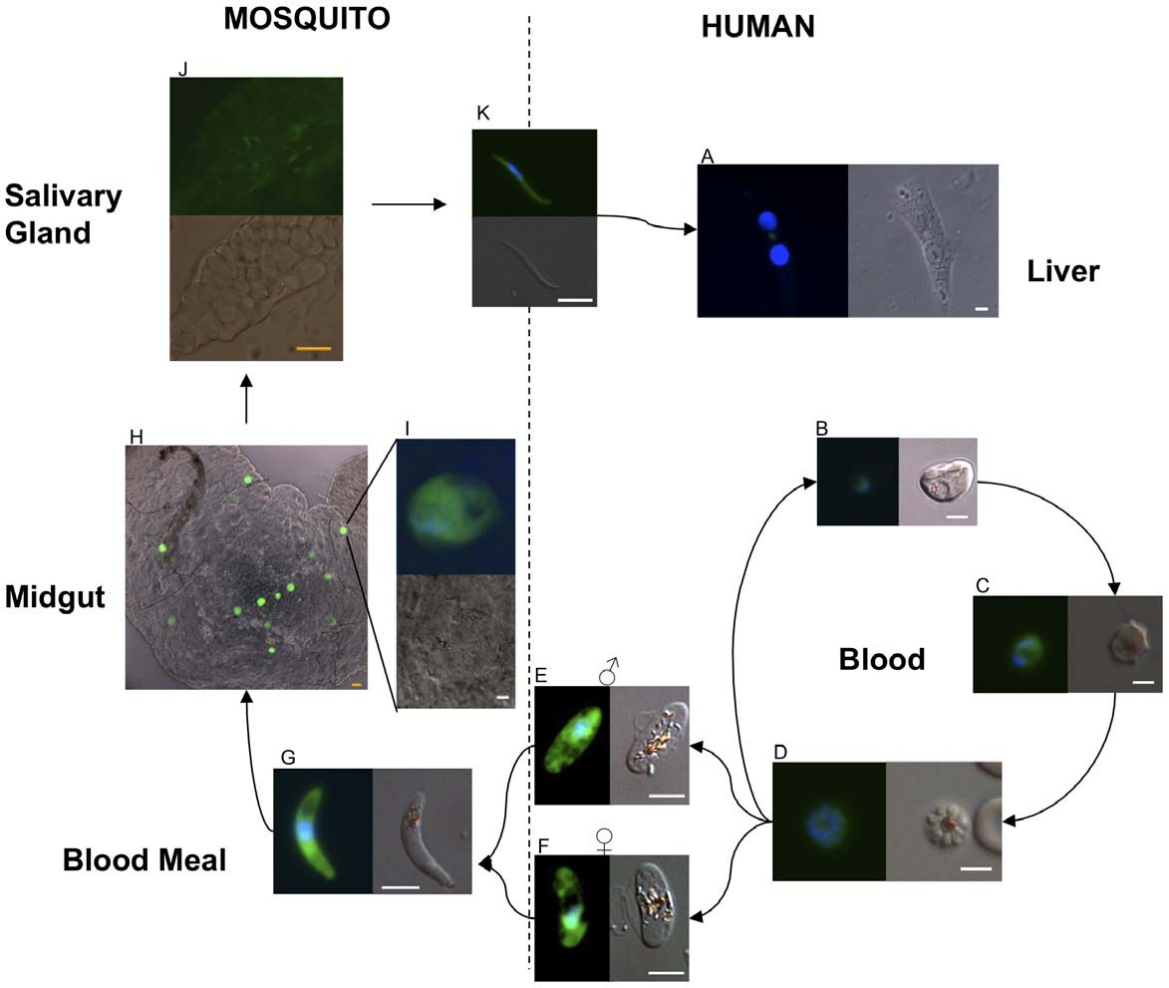
Fluorescence of 3D7HT-GFP throughout the life cycle. GFP fluorescence was observed in different life stages of the parasite. Parasites were counterstained with DAPI nuclear dye (blue) (VectorLabs, UK). Fluorescent and DIC images are shown alongside each other, or as an overlay. (A) liver stage trophozoites in a primary human hepatocyte (B) blood ring stage, (C) trophozoites, (D) schizont, (E) male gametocyte, (F) female gametocyte, (G) ookinete obtained 29 hours post-feed in infected *An. stephensi* blood meal, (H) an infected gut 8 days post-feed, (I) 10 day oocyst, (J) infected salivary gland dissected 18 days post-feed, (K) salivary gland sporozoite. White scale bar 5 µm, orange scale bar 50 µm.

3D7HT-GFP life cycle stages can be visualized in live and fixed assay in both human and mosquito tissues. Trophozoites and female gametocytes exhibited the strongest fluorescence (Fig. 3), as reported previously in *P. berghei* [2].

Studies on the invasive sporogonic forms of the parasite can especially benefit from this parasite line. Indeed *P. falciparum* ookinetes and sporozoites have previously been difficult to obtain and track *in vivo*. With our reporter parasite we were able reliably to image ookinetes and sporozoite motility in mosquito tissues (see Supplementary Movies S1 and S2). Infection of mosquito midguts and salivary glands and human hepatocytes were readily observed at low magnification (Fig. 3).

The availability of this line will further allow the reliable, rapid and simple quantification of *P. falciparum* in all stages of the life cycle, just as the extensive use of reporter strain in rodent models have previously shown [1]. This is key to a deeper understanding of host, parasite and mosquito factors that participate in transmission of the parasite from host to vector and vector to host. It will also allow for establishment of high-throughput assays examining the effect of drugs and antibodies on those processes (Delves M, personal communication).

Other applications, which have been used in rodent models, such as sorting parasites by flow cytometry (Fig. 2), *in vivo* imaging of ookinete behavior (Supplementary Mov. 1) and *ex vivo* observation of sporozoite motility (Supplementary Mov. 2) traversal and invasion are now possible.

3D7HT-GFP has been deposited at the Malaria Research and Reference Reagent Resource Center (MR4).

## Methods

### Ethics Statement

Blood products were obtained from the National Blood Service (UK). Ethical approval was obtained by the Imperial College Research Ethics Committee.

### Generation of pEF-GFP

An 800 bp fragment of the *P. falciparum* EF1α (PF13_0304) promoter region was amplified with primers 5′- AATAGATCTTTTAACGGTTCACCCCTCTTAACC-3′ (*BglII* site underlined) and 5′- AATAGGTACCGTTTAGTATATTAATATATATGTATA-3′ (*KpnI* site underlined) and was inserted into pARL vector. A 900 bp homology region of the *Pf47* coding sequence (PF13_0248) was next amplified with primers 5′- GGGGCGCCGAATCTCATTTATATTCTGC -3′ (*SfoI* site underlined) and 5′- GGGGCGCCTAACATATACATGCCTTCC -3′ (*SfoI* site underlined) and inserted it into the plasmid, thus obtaining pEFGFP (Fig. 1). PCRs were carried out with Advantage Taq II Polymerase (Takara Bioscience, UK), restriction enzymes were obtained from NEB (UK), ligation reactions were set-up with T4 DNA Ligase (Promega, UK) and transformed in XL-10 Gold ultracompetent cells (Stratagene, UK).

**Parasite Maintenance and Transfection**

Strain 3D7 was maintained in culture as described previously [13]. Briefly, parasites were cultured in 4% haematocrit RPMI 1640 (Gibco, UK), supplemented with 10% human AB serum, and gassed with 5% CO_2_, 0.5% O_2_ in N_2_ at 37°C. Parasitaemia was maintained below 5% at all times. Parasites were synchronized with the sorbitol method as described [14].

Parasites were transfected as described before [15]. Briefly, 200 µl of 5% infected red blood cells at ring stage were resuspended in 400 µl of cytomix (120 mM KCl, 0.15 mM CaCl_2_, 2 mM EGTA, 5 mM MgCl_2_, 10 mM K_2_HPO_4_/KH_2_PO_4_, 25 mM Hepes, pH 7.6) containing 80 µg of *pEFGFP*. The suspension was electroporated with a GenePulser II (BioRad, UK) (310 V and 950 µF). The electroporated parasites were immediately placed in 10 ml of complete culture media containing 120 µl of fresh red blood cells. After 8 hours, WR99210 (Jacobus Pharmaceuticals, USA) was added to the cultures to a final concentration of 5 nM. Drug-resistant parasites were subjected to successive rounds of on/off drug selection. The integration was monitored by PCR (Fig. 1).

The transfectants were genotyped by PCR (Fig. 1), cloned by limiting dilution and further genotyped by PCR and southern blotting (Fig. 1).

### Gametocyte Culture and Mosquito Infection

Gametocytes were produced as described previously [16]. Briefly, drug-resistant parasites were cultured without the addition of fresh red blood cells at 4% haematocrit. On day 16 the culture was then spun down on a cushion of 250 µl of fresh red blood cells at 600 g for 6 minutes at 37°C. The supernatant was discarded and the pellet was resuspended in an equal volume of human AB serum and fed to *Anopheles stephensi* adult females through a standard mosquito feeder. Mosquito midguts were dissected at day 1–10. Ookinetes were obtained from midguts of infected *A. stephensi* females 32–36 hours post-feed, midguts containing the blood meal were gently homogenized and resuspended in an equal volume of ice-cold Matrigel (BD Biosciences, UK). Salivary glands were processed in the same way.

All samples were observed on a DMR (Leica, UK) and acquired and analysed with the Zeiss AxioCam HRC and Axiovision software (Zeiss, UK).

### Primary Hepatocyte Infections

Primary human hepatocytes (Lonza, UK) were seeded in LabTek chamber slides (Nunc, UK) in HCM media (Lonza, UK) and kept at 5% CO2, 0.5% O2 in N2 at 37°C. Sporozoites were obtained from salivary gland of female *A. stephensi* mosquitoes 18 days after infection. 5000 sporozoites were applied to the hepatocyte cultures. Parasites were observed 24 hours after infection.

### Flow Cytometry

Asexual and gametocyte parasite cultures were pelleted at 500 g, washed twice in 2% FCS (Gibco, UK) in PBS, fixed in 2% PFA in PBS for 10 minutes at RT, and resuspended in 2% FCS (Gibco, UK) in PBS containing DRAQ5 (Cell Signalling, UK). Parasites were analyzed on a FACs Calibur 2 (BD, UK).

### Statistics

Non-parametric tests were used to compare asexual growth and gametocytaemia (Wilcoxon signed rank test) and oocyst counts (Mann- Whitney test). These analyses were conducted with GraphPad Prism (GraphPad Software, USA).

## Supporting Information

Movie S1 3D7HT-GFP ookinete exhibiting gliding behaviour. An ex vivo ookinete was obtained from an Anopheles stephensi infected bloodmeal 32 hours post-feeding, resuspended in Matrigel (BD Bioscience, UK) and imaged for 10 minutes. Scale bar 10 µm.
(1.74 MB MOV)

Movie S2 3D7HT-GFP sporozoites exhibiting gliding behaviour. A salivary gland was obtained from an Anopheles stephensi 18 days post-infection, and resuspended in Matrigel (BD Bioscience, UK) and imaged for 6 minutes. Scale bar 20 µm.
(3.20 MB MOV)
